# Preparation of Nanoparticles Loaded with Membrane-Impermeable Peptide AC3-I and Its Protective Effect on Myocardial Ischemia and Reperfusion

**DOI:** 10.3390/pharmaceutics16030416

**Published:** 2024-03-18

**Authors:** Yi Liu, Yingyi Niu, Wenjie Zhang, Kaikai Wang, Tianqing Liu, Weizhong Zhu

**Affiliations:** 1 School of Pharmacy, Nantong University, Nantong 226001, Chinakwang@ntu.edu.cn (K.W.); 2 NICM Health Research Institute, Western Sydney University, Sydney, NSW 2145, Australia

**Keywords:** CaMKII, AC3-I, nanoparticle, cardiomyocytes, ischemia–reperfusion injury

## Abstract

Purpose: It is well known that inhibition of Ca^2+^/calmodulin-dependent protein kinase II (CaMKII) provides cardiac protection in cases of myocardial ischemia–reperfusion injury. However, there are currently no cytoplasm-impermeable drugs that target CaMKII. The aim of this study was to develop curcumin albumin nanoparticles (HSA-CCM NPs) containing AC3-I and investigate their protective effects on hypoxia–reoxygenation (H/R)-induced injuries in adult rat cardiomyocytes and ischemia–reperfusion (I/R) injuries in isolated rat hearts. Methods: HSA-CCM NPs were synthesized using β-ME methods, while the membrane-impermeable peptide AC3-I was covalently linked via a disulfide bond to synthesize AC3-I@HSA-CCM NPs (AC3-I@NPs). Nanoparticle stability and drug release were characterized. To assess the cardiomyocyte uptake of AC3-I@NPs, AC3-I@NPs were incubated with cardiomyocytes under normoxia and hypoxia, respectively. The cardioprotective effect of AC3-I@NPs was determined by using a lactate dehydrogenase kit (LDH) and PI/Hoechst staining. The phosphorylation of phospholamban (p-PLB) was detected by Western blotting in hypoxia–reoxygenation and electric field stimulation models. To further investigate the protective role of AC3-I@NPs against myocardial ischemia–reperfusion injury, we collected coronary effluents and measured creatine kinase (CK) and LDH release in Langendorff rat hearts. Results:AC3-I@NPs were successfully prepared and characterized. Both HSA-CCM NPs and AC3-I@NPs were taken up by cardiomyocytes. AC3-I@NPs protected cardiomyocytes from injury caused by hypoxia–reoxygenation, as demonstrated by decreased cardiomyocyte death and LDH release. AC3-I@NPs reduced p-PLB levels evoked by hypoxia–reoxygenation and electrical field stimulation in adult rat cardiac myocytes. AC3-I@NPs decreased the release of LDH and CK from coronary effluents. Conclusions: AC3-I@NPs showed protective effects against myocardial injuries induced by hypoxia–reoxygenation in cardiomyocytes and ischemia–reperfusion in isolated hearts.

## 1. Introduction

Despite numerous clinical practice interventions aiming to reduce cardiac remodeling and failure caused by myocardial ischemia or reperfusion injury after myocardial infarction, the incidence of heart failure and related cardiovascular events such as hospitalization after myocardial infarction persists [1]. Therefore, finding efficient medications to prevent myocardial ischemia is crucial. Though the exact causes of ischemia–reperfusion injury are still unknown, a number of factors, including calcium overload, mitochondrial dysfunction, free radicals, reactive oxygen species, inflammation, and neutrophil-mediated vascular injury, are presently recognized as potential contributors [2,3]. In addition, drugs that target those of pathophysiological process can only achieve limited therapeutic outcomes for the treatment of myocardial ischemia–reperfusion injury due to their low stability, low bioavailability, short half-life, and side effects. Therefore, it is vital to develop effective drugs and technologies to address this issue. Nanotherapies have demonstrated great opportunities to treat myocardial ischemia–reperfusion damage [4,5]. Because of their unique size, shape, and material functions, nanomedicines can effectively increase the therapeutic impact and reduce adverse effects by improving pharmacokinetic and pharmacodynamic properties when compared to traditional drugs.

Ca^2+^/calmodulin-dependent protein kinase II (CaMKII) plays an important physiological role in the regulation of cardiac excitatory contraction coupling [6]. Furthermore, CaMKII is essential for the death response of myocardial cells and cardiac remodeling post I/R injury [7]. CaMKII is usually activated by high concentrations of intracellular calcium ions, whereas new research has indicated that its activity is also regulated by reactive oxygen species (ROS) oxidation [8,9] and glycosylation modification [10]. In fact, the activation of CaMKII is a common intermediate product of various death stimuli that induce myocardial cell apoptosis [11,12]. Thus, CaMKII is considered as a possible target for ischemia–reperfusion injury. However, a cellular membrane-permeable and specific inhibitor of CaMKII is not available yet.

Although KN-93 is the most widely used CaMKII inhibitor for studying the cellular and in vivo activities of CaMKII [13], it is commonly believed that KN-93 binds directly to CaMKII, thus blocking kinase activation by competing with Ca^2+^/CaM. According to a recent study [14], KN-93 binds directly to Ca^2+^/CaM rather than CaMKII, which impairs the ability of Ca^2+^/CaM to interact with CaMKII and, hence, indirectly inhibits CaMKII activation. This Ca^2+^/CaM-dependent, non-CaMKII activity should be taken into account in KN-93-based mechanism of action investigations and drug discovery efforts [14]. A recent screen against 234 protein kinases showed that KN-93 is extremely selective, while its targets include Fyn, Haspin, Hck, Lck, MLCK, Tec, and TrkA [15]. In addition to having direct effects on the channel, KN-62 and KN-93 also prevent CaMKII from modulating the L-type Ca^2+^ channel [16]. Autocamted-3 inhibitor (AC3-I) and autocamted-2 inhibitor protein (AIP) are substrate-based inhibitors. The modification of self-inhibitory regulatory fragments to inhibit the activation of CaMKII promotes the development of prolonged inhibitory peptides missing CaM binding sequences (residues 273–302). As demonstrated by animal experiments, AC3-I and AIP, as inhibitors of CaMKII, can influence CaMKII activity by genetically engineering the expression of AC3-I in vivo and providing protective effects against cardiomyopathies such as cardiac remodeling. Although their selectivity has not been widely analyzed, they are more than 100 times more effective in selectively inhibiting CaMKII than PKC, PKA, and CaMKIV, respectively, despite the fact that their selectivity has not been well examined [13]. However, the in vivo application of AC3-I is restricted due to its impermeability of the cell membrane.

It has been proved that curcumin has protective effects on myocardial ischemia–reperfusion. The possible mechanism involves the regulation of the expression of inflammatory factor and apoptotic factor TRL4 and scavenging oxygen free radicals against oxidative damage [17].

There are 17 intramolecular disulfide bonds in the tertiary structure of HSA [18]. HSA is rich in sulfhydryl groups, accounting for more than 80% of the total sulfhydryl groups in plasma, which can remove reactive oxygen species and nitrogen. In addition, it carries substances such as nitric oxide and bilirubin and provides additional protection against oxidative stress. There is evidence that HSA can inhibit coagulation and platelet aggregation. Its colloid permeation effect and its ability to interact with endothelial glycocalyx contribute to the stability of the capillary membrane and the liquid balance of the capillary wall.

The biodistribution and therapeutic performance of drugs can be improved by encapsulating drugs using nanotechnology and developing new nanoparticle formulations. Nanomedicines can target the infarcted heart tissue passively or actively [19]. Passive targeting is based on the enhancement of cardiac permeability and retention effects (EPR) after myocardial infarction, as well as the use or avoidance of recognition by the reticular endothelial system (RES) to achieve targeted effects [20]. Conversely, active targeting relies on the anatomical and functional characteristics of the targeted organ. Properly designed and prepared nanocarriers can selectively distribute in the target organ without damaging surrounding normal cells, efficiently deliver drugs to diseased cells or tissues, and accumulate at the desired site [21].

In this study, curcumin albumin nanoparticles were synthesized, and the disulfide bonds were opened by DTT to form active sulfhydryl groups. AC3-I@NPs were synthesized by reacting AC3-I with the sulfhydryl L-cysteine of AC3-I to form disulfide bonds.

Therefore, this study aimed to construct an advanced nanoparticle formulation that targets damaged myocardium during myocardial ischemia–reperfusion and carries the membrane-impermeable peptide AC3-I. We also aimed to investigate their protective effect on myocardial ischemia–reperfusion and the mechanism of action via reducing cell CaMKII activity.

## 2. Materials and Methods

### 2.1. Animals

Male Sprague Dawley (SD) adult rats weighing 250–280 g were selected, all of which were provided by the animal experimental center of Nantong University. This study was approved by the experimental animal management and use committee of Nantong University. The production license number is SYXK9 (Su) 2007-0021.

### 2.2. Reagents

AC3-I@NPs were made by the sulfhydryl reaction of albumin with the L-Cysteine of AC3-I (KKALHRQEAVDCL) to form disulfide bonds. Phospholamban (Santa Cruz Biotechnology, Dallas, TX, USA) and Tubulin (Aibixin Biotechnology Co., Ltd., Beijing, China) were used. Creatine kinase-MB (CK-MB) was detected using commercial kits from the Nanjing Jiancheng Bioengineering Institute, Nanjing, China. Lactate dehydrogenase (LDH), propidium iodide (PI), and Hoechst 33342 were purchased from Beyotime of Nantong, China.

### 2.3. Preparation of Curcumin Albumin Nanoparticles

At 37 °C, HSA (100 mg) was dissolved in 50 mL of Tris buffer (pH 7.4), and β-ME (5 mM) was added to the solution and stirred for 10 min. Subsequently, CCM (20 mg in DMSO as solvent) was added. The HSA-CCM NPs were dialyzed (molecular weight: 10 kD) for 24 h. They were prefrozen in a refrigerator with a temperature of −80 °C and then freeze-dried in a freeze-dryer.

### 2.4. Preparation of AC3-I@NPs

The HSA-CCM NPs (5 mg) were dissolved in 5 mL of DDH_2_O, and 10 μL of DTT solution (5 mg/mL) was added. After 10 min of mixing, 0.75 mg of AC3-I was added and incubated at 37 °C for 30 min.

### 2.5. Morphology, Particle Size, and UV-Vis Spectral Analysis of AC3-I@NPs

A drop of AC3-I@NPs was placed on a carbon-coated grid. After the samples were dried, the AC3-I@NPs were subjected to transmission electron microscopy at an accelerated voltage of 200 kV. Using dynamic light scattering, the size distribution of the nanoparticles was measured. For each sample, the analysis was run 5 times, with the acquisition time being set to 30 s for each run. The absorption of the HSA solution was measured using a UV-Vis 1700 spectrophotometer (Shanghai Metash Instruments Co., Ltd., Shanghai, China) to record the absorption spectra.

### 2.6. Release of CCM in AC3-I@NPs

The in vitro drug release of CCM in nanoparticles (AC3-I@NPs, HSA-CCM NPs) was measured in phosphate-buffered saline (PBS, 0.15 M, pH 7.4) containing 2% (*v*/*v*) Tween 80. The nanoparticle solutions (2 mg/mL) were placed into dialysis tubes (molecular weight = 12,000). The dialysis tubes were completely immersed in 20 mL release medium and stored at 100 rpm in a 37 °C incubator. At a predetermined time interval, 2 mL of the release medium was retained and replaced with an equal amount of fresh release medium to maintain the settling conditions. The concentration of CCM was determined using a UV-Vis spectrophotometer.

### 2.7. Stability of AC3-I@NPs in Solution

To investigate the stability of the nanoparticles, the AC3-I@NPs were dispersed in pH 7.4 phosphate-buffered saline (PBS) for 2 weeks. The solution was stored at 4 °C, and the diameter of the fluid dynamics was measured at different times using dynamic light scattering (DLS).

### 2.8. Isolation and Culture of Adult Rat Cardiac Myocytes

After the rats were anesthetized, their hearts were quickly removed. After draining the remaining blood from the heart, it was quickly hooked up to a digestive device. The heart tissue was digested by type II collagenase and proteinase. After digestion, the ventricle was cut off, the tissue was cut to pieces, digested on a shaking table, and sifted through a 100-mesh sieve to assess the gradient deposition of the cardiomyocytes. The isolated cardiomyocytes were cultured in Medium 199 (M199, Sigma, Livonia, MI, USA) supplemented with 5 mM creatine, 5 mM taurine, and 2 mM carnitine for 2–4 h.

### 2.9. Hypoxia–Reoxygenation (H/R) of Cardiac Myocytes

Fresh isolated adult rat cardiomyocytes (ARCMs) were attached to culture dishes pretreated with Laminin (10 mg/mL of PBS). The M199 medium was changed and then cultured for 2 h under normal oxygen conditions for subsequent experiments. Then, the ARCMs were maintained in an anaerobic incubator (95% N_2_ and 5% CO_2_) at 37 °C for 8 h in the presence of the AC3-I@NPs or different designed groups. The cells were then cultured for 9 h or reoxygenated in a 37 °C incubator (95% air and 5% CO_2_). The normoxia control group was exposed to 95% air and 5% CO_2_ for 17 h.

### 2.10. PI/Hoechst Staining

The original medium in the plate was discarded after treatment, and the cells were covered with 250 μL of Hoechst staining solution and cultured in the incubator for 25 min. The cells were washed three times with PBS. PI staining solution diluted with M199 medium was added and incubated for 25 min. After cell staining, pictures were taken using a Leica microscope.

### 2.11. Measurement of Lactate Dehydrogenase (LDH)

The medium was extracted from the plate and centrifuged at 400 rpm for 5 min. The medium supernatants from different groups were added into a new 96-well plate, and then 60 mL of LDH working fluid was added into the medium. The optical density (OD) was measured spectrophotometrically at 490 nm using an enzyme meter.

### 2.12. Electrical Field Stimulation in Primary Adult Rat Cardiomyocyte

Adult rat cardiomyocytes were isolated, rod-shape myocytes were purified until 90% purity, and the cell suspension was placed in the incubator. The cell suspension was placed on the electrical field plates. The cell suspension was stimulated (2 Hz) for 5 min at 20 V and 0.6 ms, respectively, and then the protein was rapidly extracted in the presence of a phosphatase inhibitor. During stimulation, the beating of cardiac myocytes was observed under a microscope to confirm the cellular electrical responses.

### 2.13. Western Blot

Proteins were extracted from the isolated ARCMs, determined using the BCA Detection Kit (Beyotime Biotechnology, Shanghai, China), and separated by 12% sodium lauryl sulfate–polyacrylamide gel electrophoresis before being imprinted onto polyvinylidene fluoride membranes (Millipore, Billerica, MA, USA). After blocking in 5% skim milk, they were co-incubated with primary antibodies (1:1000) and incubated at 4 °C overnight. After washing with Tris-buffered saline and Tween 20 (TBST), the membranes were incubated with secondary antibodies for 2 h at room temperature. After washing, the immune complex was visualized by enhanced chemiluminescence, and the band strength was measured quantitatively using Image j software (Version 1.8.0.112, NIH, Bethesda, MD, USA).

### 2.14. Langendorff-Isolated Adult Rat Heart Perfusion

A Krebs–Henseleit (K-H) buffer (in mM) containing 131 NaCl, 4.0 KCl, 1.2 KH_2_PO_4_, 1.2 MgSO_4_, 1.8 CaCl_2_, 25.0 NaHCO_3_, and 11.0 glucose was prepared, and equilibrated at 37 °C with a mixture of gases (95% O_2_ and 5% CO_2_) to pH 7.35–7.45. The rats were anesthetized with sodium pentobarbital (40 mg/kg), and their hearts were removed and drained of residual blood. The hearts were placed in a Langendorff system at room temperature. The coronary outflow was collected at designated time points.

### 2.15. Experimental Groups and Treatments

Twenty rats were randomly divided into 4 groups: (1) the normoxia group; (2) the ischemia–reperfusion group, the procedure for which consisted of 50 min equilibration under conditions of 95% N_2_ and 5% CO_2_ and 40 min reperfusion with glucose-containing K-H buffer; (3) the AC3-I@NPs group, the procedure for which consisted of 20 min equilibration, heart perfusion with K-H buffer containing AC3-I@NPs (5 µM) for 20 min, and the ischemia–reperfusion procedure; and (4) the AIP group, the procedure for which had similar experimental steps to that of (3) but involved the use of 5 µM AIP in the treatment.

### 2.16. Detection of Biochemical Indexes

Coronary outflow was collected within 120 min after reperfusion at designated time points. Myocardial tissue enzyme LDH and creatine kinase MB levels in coronary effluents were measured using commercial kits to assess cardiac damage according to the manufacturer’s instructions.

### 2.17. Statistical Analysis

Data are presented as the Mean ± S.E.M of three independent experiments. Statistical analysis was performed using GraphPad Prism version 8.0.1. The results were compared using a one-way ANOVA followed by Dunnett’s test. *p* < 0.05 was considered to represent a statistically significant difference.

## 3. Results

### 3.1. Characterization of AC3-I@NPs

**Figure 1:**
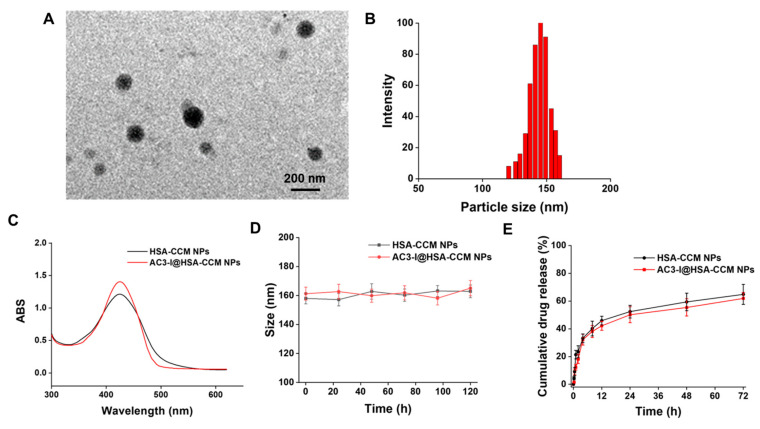
Characterization of AC3-I@NPs. (**A**) TEM image of dry AC3-I@NPs on an aluminum grid. The scale is 200 nanometers. (**B**) Histogram of size distribution of AC3-I@NPs. (**C**) UV-Vis absorption spectra of HSA-CCM NPs and AC3-I@NPs. (**D**) In vitro stability of HSA-CCM NPs and AC3-I@NPs in PBS at 37 °C, pH 7.4. (**E**) Release of CCM in HSA-CCM NPs and AC3-I@NPs.

The morphology and particle size of AC3-I@NPs. The disulfide bonds of albumin were opened with DTT to obtain active thiol groups. AC3-I@NPs were synthesized by the formation of a disulfide bond between the activated thiol group with the thiol group of cysteine in AC3-I. After the preparation of AC3-I@NPs, the morphology and size of the AC3-I@NPs were observed using transmission electron microscopy (TEM). As shown in Figure 1A, the nanoparticles have a spherical morphology and smooth surface. The TEM images were used to determine the nanoparticle size distribution. As illustrated in Figure 1B, the average particle size of the AC3-I@NPs is approximately 156 nm.

UV-Vis absorption spectra, in vitro stability, and release properties of AC3-I@NPs. The HSA-CCM NPs and AC3-I@NPs were analyzed by measuring their UV-Vis absorption spectra, respectively (Figure 1C). According to the UV-Vis spectra results, the characteristic absorption peaks of curcumin appeared at 422 nm after full-wavelength scanning. The in vitro stability and drug release of nanoparticle were studied (Figure 1D,E). In PBS at 37 °C and pH 7.4, the two groups of nanoparticles were stable with a particle size of around 160 nm. The release rate regarding the release of CCM from the AC3-I@NPs within 72 h was 61.88 ± 4.27%, while the release rate of the HSA-CCM NPs within 72 h was 64.78 ± 7.18%. The above characterization results suggest that we successfully prepared AC3-I@NPs.

### 3.2. Uptake of AC3-I@NPs in Cultured Rat Cardiomyocytes

**Figure 2:**
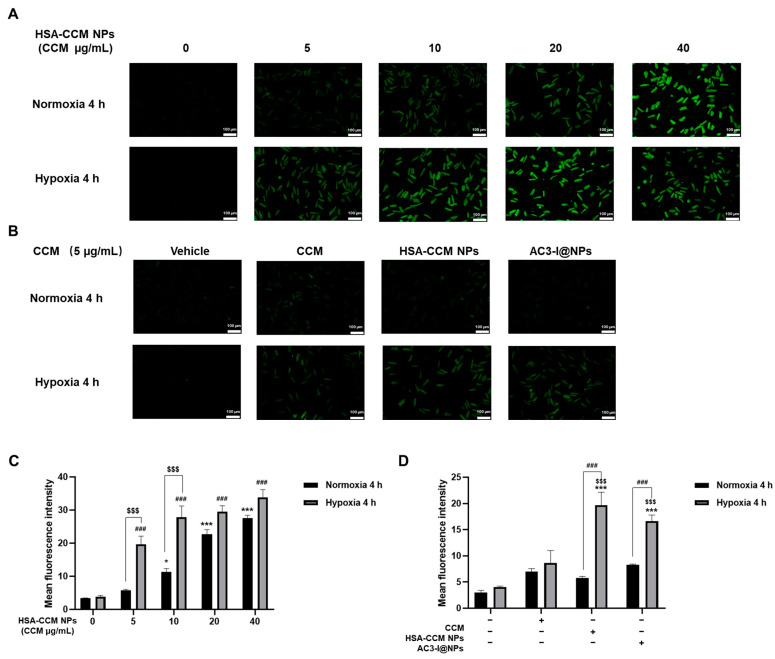
HSA-CCM NPs and AC3-I@NPs were taken up by cardiomyocytes under normoxia and hypoxia. (**A**,**B**) Fluorescent images of cellular uptake results. (**C**) Quantitative measurements of the cellular uptake of HSA-CCM NPs by cardiomyocytes under normoxia or hypoxia for 4 h. (**D**) Quantitative measurements of the cellular uptake of CCM, HSA-CCM NPs, AC3-I@NPs by cardiomyocytes cultured in normoxia or hypoxia for 4 h. Comparison of normoxia control group, * *p* < 0.05, *** *p* < 0.001; comparison of hypoxia control group, ^###^ *p* < 0.001; normoxia group compared with hypoxia group, ^$$$^ *p* < 0.001. Comparison of hypoxia group, *** *p* < 0.001; comparison of CCM group, ^$$$^ *p* < 0.001; normoxia group compared with hypoxia group, ^###^ *p* < 0.001. *n* = 3, Mean ± S.E.M.

We investigated whether HSA-CCM NPs could be taken up by cultured rat cardiac myocytes. The curcumin concentrations in the HSA-CCM NP groups were 5, 10, 20, and 40 µg/mL, respectively. Under normal oxygen and hypoxia conditions, curcumin fluorescence was used to monitor the fluorescence intensity of cardiac myocytes under a fluorescence microscope, as shown in Figure 2A. More notably, cellular uptake was significantly increased following hypoxia compared to normoxia. It was also observed that under hypoxia conditions, when the concentration of HSA-CCM NPs increased, the uptake of nanoparticles by cardiomyocytes gradually reached saturation (Figure 2C).

In addition, to investigate whether cardiac myocytes can still take up membrane-impermeable-loaded nanoparticles (AC3-I@NPs), we prepared AC3-I@NPs by setting the AC3-I concentration to 10 μM and the concentration of curcumin to 5 μg/mL. In addition, the concentrations of curcumin in the CCM and HSA-CCM NPs were consistent with that of the AC3-I@NPs. Under normal and hypoxic conditions, each group was co-incubated with cardiac myocytes for 4 h, and the fluorescence intensity of myocardial cells was recorded using a microscope, as shown in Figure 2B. The results indicated that AC3-I@NPs were taken up by cardiac myocytes after loading AC3-I. Compared with the normoxia treatment, significantly more AC3-I@NPs were accumulated in cardiomyocytes under hypoxia (Figure 2D).

### 3.3. Biocompatibility of AC3-I@NPs

**Figure 3:**
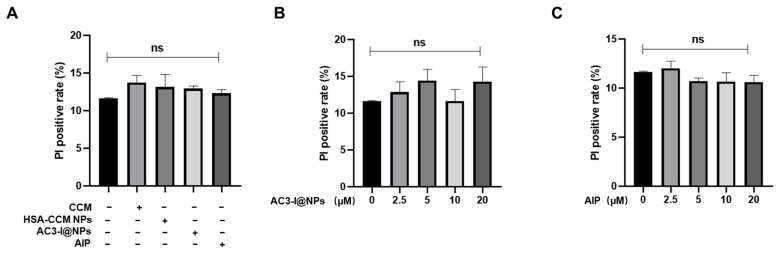
AC3-I@NPs had no cytotoxicity to cardiomyocytes. (**A**) Cytotoxic effects of each group on cardiomyocytes at 10 μM. (**B**) Toxic effects of different concentrations of AC3-I@NPs on cardiomyocytes. (**C**) Effect of different concentrations of AIP on myocardial cell toxicity. Compared with the blank control group, there was no significant difference.

To verify whether the nanoparticles were biocompatible, the cardiomyocytes were treated with the AC3-I@NP, HSA-CCM NP, AIP, and CCM groups for 17 h. As shown in Figure 3A, the concentration of AC3-I in NP and AIP was 10 μM. The dose of HSA-CCM-NP as a vehicle and the dose of CCM were equal to that in the AC3-I@NPs. Cell viability was measured using a fluorescence microscope after the cells were stained with PI/Hoechst, and the cell mortality rate was calculated, as shown in Figure 3A. Our data indicate that the AC3-I@NPs had no harmful effects on the cultured rat adult cardiac myocytes. Moreover, the cardiomyocytes were treated with AC3-I@NPs and AIP at varying doses (2.5, 5, 10, 20 µM) for 17 h. As shown in Figure 3B,C, all concentrations of the AC3-I@NPs and AIP had no toxic effects on the cardiac myocytes.

### 3.4. AC3-I@NPs Reduced Cell Death Caused by Hypoxia and Reoxygenation

**Figure 4:**
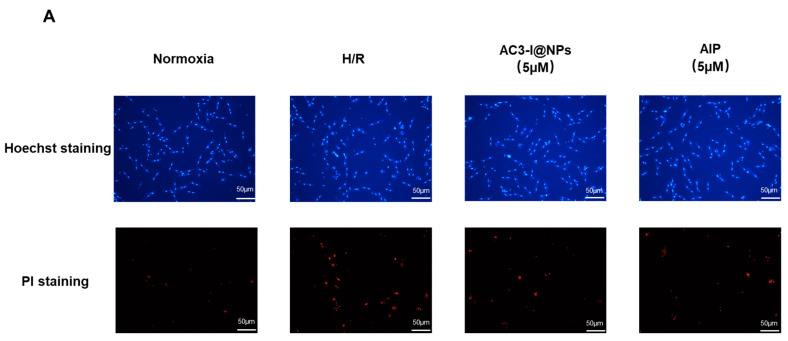

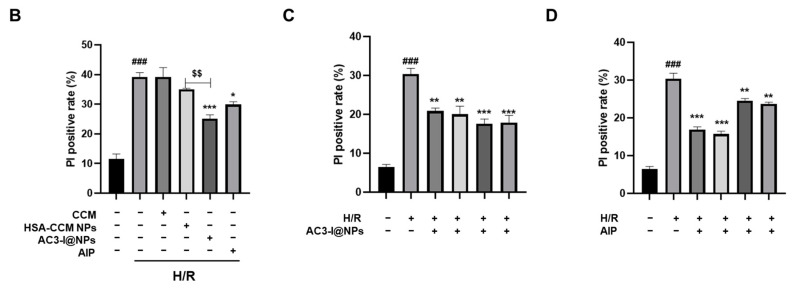
AC3-I@NPs reduced cell death induced by hypoxia–reoxygenation. (**A**) Plot representative of the PI/Hoechst staining of AC3-I@NPs and AIP. (**B**) All experimental groups reduced H/R-induced cardiomyocyte death. (**C**) AC3-I@NPs reduced H/R-induced cardiomyocyte death. (**D**) AIP reduced H/R-induced cardiomyocyte death. Compared with the normoxia group, ^###^ *p* < 0.001; compared with H/R group, * *p* < 0.05, ** *p* < 0.01, *** *p* < 0.001; compared with HSA-CCM NPs, ^$$^ *p* < 0.01. n = 3, Mean ± S.E.M.

To explore whether AC3-I@NPs could alleviate cell damage caused by hypoxia–reoxygenation, the cells were treated with the corresponding groups and cultured under hypoxia for 8 h and reoxygenation for 9 h. The myocardial cell mortality rate was calculated using PI/Hoechst staining, as shown in Figure 4B–D. The results demonstrate that the AC3-I@NPs and AIP reduced cardiomyocyte death. In addition, the AC3-I@NPs significantly alleviated the damage induced by hypoxia and reoxygenation compared to the HSA-CCM NPs.

### 3.5. AC3-I@NPs Alleviated the Cell Damage Caused by Hypoxia and Reoxygenation

**Figure 5:**
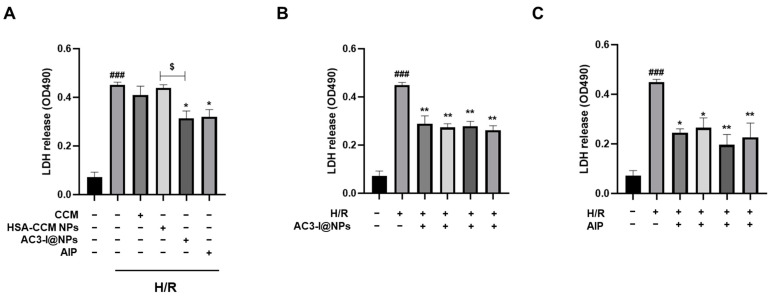
AC3-I@NPs decreased LDH release after hypoxia–reoxygenation induction. (**A**) H/R-induced LDH release was decreased in all experimental groups. (**B**) AC3-I@NPs reduced H/R-induced cardiomyocyte LDH release. (**C**) AIP reduced H/R-induced LDH release from cardiomyocytes. Compared with normoxia group, ^###^ *p* < 0.001; compared with H/R group, * *p* < 0.05, ** *p* < 0.01; compared with HSA-CCM NPs, ^$^ *p* < 0.05. n = 3, Mean ± S.E.M.

To further investigate the possibility of the AC3-I@NPs shielding the cells from damage induced by hypoxia–reoxygenation, the cells were treated and cultured under 8 h of hypoxia and 9 h of reoxygenation. The lactate dehydrogenase content in each group was detected by using an LDH reagent kit. The findings demonstrate that the LDH release content in the H/R group was substantially higher than that of the blank control group. Furthermore, the AC3-I@NPs significantly alleviated cell damage compared to the HSA-CCM NPs. More importantly, at every dose of AC3-1 (2.5 to 20 μM of AC3-I), the AC3-I@NPs were able to reduce cell damage induced by hypoxia and reoxygenation, as illustrated in Figure 5.

### 3.6. AC3-I@NPs Reduced p-PLB Levels Evoked by Hypoxia–Reoxygenation and Electrical Field Stimulation in Adult Rat Cardiac Myocytes

**Figure 6:**
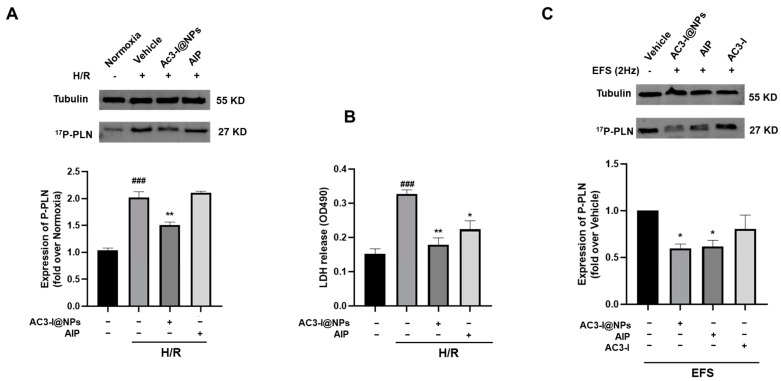
AC3-I@NPs reduced the expression of P-PLB to reduce CAMKII activity. (**A**) Western blot analysis for the expression of p-PLB. (**B**) LDH released from the supernatants of each group. (**C**) Western blot analysis for the expression of p-PLB under 2 Hz stimulation. Compared with the normoxia group, ^###^ *p* < 0.001; compared with the H/R group, * *p* < 0.05, ** *p* < 0.01; compared with the blank control group, * *p* < 0.05. n = 3, Mean ± S.E.M.

To study whether the AC3-I@NPs could inhibit the activation of CaMKII, the expression of phosphorylated phospholamban (p-PLB), an intrinsic substance of CaMKII in cardiomyocytes, was analyzed using Western blotting in an electrical field stimulation (EFS)-evoked model. In the presence of 5 µM of AC3-I@NPs and with the use of the AIP group as a positive control, myocytes were cultured under hypoxia for 8 h and reoxygenation for 9 h. Western blotting was employed to detect the change in p-PLB. At the same time, the supernatant was aspirated, and the lactate dehydrogenase content was detected using an LDH assay kit. Figure 6A demonstrates that the H/R group had a much higher p-PLB expression level, while AC3-I@NPs reduced the p-PLB expression level compared to the blank control group (Figure 6A). Accordingly, the AC3-I@NPs reduced the LDH level (Figure 6B). The inhibition of CaMKII activation by the AC3-I@NPs was confirmed in the EFS-evoked model, as shown in Figure 6C. The AC3-I@NPs reduced the intracellular CaMKII activity induced by 2 Hz of EFS in myocardial cells.

### 3.7. AC3-I@NPs Alleviated Levels of Lactate Dehydrogenase and Creatine Kinase in Efflux in Isolated Working Hearts after Ischemia–Reperfusion Injury

**Figure 7:**
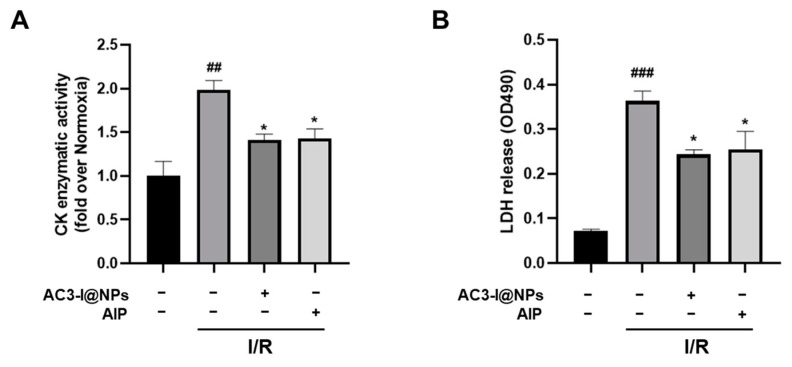
AC3-I@NPs alleviated the injuries of cardiac marker enzymes. (**A**) The change in CK content in the hearts of each group. (**B**) The change in LDH levels in the hearts of each group. Compared with the normoxia group, ^##^ *p* < 0.01, ^###^ *p* < 0.001; compared with the I/R Group, * *p* < 0.05. n = 3, Mean ± S.E.M.

To verify the protective effects of AC3-I@NPs against cardiac injury in tissues, the level of lactate dehydrogenase and creatine kinase in the collected coronary outflows of the isolated hearts were measured using LDH and CK kits in the presence of 5 µM of AC3-I@NPs and AIP, respectively. The results showed that compared with the normoxia group, the LDH and CK levels were significantly increased in the isolated hearts of the I/R group. More importantly, both the AC3-I@NPs and AIP were able to reduce LDH and CK levels, as shown in Figure 7.

## 4. Discussion

Despite the numerous clinical practice interventions aiming to reduce cardiac remodeling and failure caused by myocardial ischemia or reperfusion injury after myocardial infarction, the incidence of heart failure and related cardiovascular events, such as hospitalization after myocardial infarction, persists. Therefore, finding efficient medications to prevent myocardial ischemia is crucial. In the present study, AC3-I@NPs were successfully prepared and showed cardiac-protective effects.

CaMKII, a multifunctional serine/threonine protein kinase, is particularly important in cardiac physiology and pathophysiology. It is commonly understood that the early and rapid recovery of ischemic-site blood flow is essential for ischemic myocardium repair and for preventing further damage after myocardial ischemia. However, in fact, the process of reperfusion can lead to additional myocardial damage, arrhythmia, and systolic dysfunction [22]. While myocardial cells are still alive at the beginning of reperfusion, they begin to die during the process of blood flow recovery due to ROS accumulation, calcium overload, and inflammation [23,24]. Therefore, in order to alleviate reperfusion injury, some nanoformulations targeting ROS sources are currently being developed. CaMKII is an ROS-regulated protein kinase. CaMKII inhibition strategies have shown great potential for the treatment of heart failure or arrhythmia [25,26]. Animal experiments have demonstrated that myocardial cells expressing the specific CaMKII inhibitory peptide AC3-I, the same amino acid sequence as that in the present study, can weaken cardiac remodeling and the degree of heart failure. Therefore, CaMKII is becoming more widely acknowledged as an important target protein for drug interventions in heart diseases. However, there is still a lack of compounds that are suitable for humans [13]. The present study developed AC3-I-based nanomedicines targeting CaMKII, and these nanomedicines displayed good stability and physicochemical properties and had excellent cardiac protection functions.

Curcumin (CCM) is a natural polyphenol molecule extracted from Curcuma longa. It numerous significant therapeutic benefits, such as its antioxidant, anti-inflammatory, anti-cancer, anti-viral and anti-microbial, anti-arthritis, and other properties, have been demonstrated [27]. In this study, it was used as a fluorescent probe, and it also had a synergistic effect with AC3-I when the nanomedicines were designed. This current study, however, did not show any obvious cytoprotection in the experimental conditions, which may be related to the curcumin dosage we used.

As the most abundant protein component in plasma, human serum albumin (HSA) has been proved to be immunogenicity-free, biocompatible, biodegradable, and metabolizable in vivo [28]. HSA plays many important roles in the advancement of drug therapy. Because of its unique molecular structure and inherent ability to bind with drug molecules, HSA can be directly utilized as a monomer formula or made into a protein-based nanoparticle platform to achieve the targeted delivery of therapeutic molecules. In addition, HSA can also be employed as a protein stabilizer or environmental response material to hybridize with functional materials, including polymers or inorganic nanoparticles, through a covalent reaction or electrostatic interaction. This can significantly alter the biological distribution and pharmacokinetic behavior of the drug, improving its therapeutic efficacy. Extensive research has been conducted to develop therapeutic drug agents conjugated with HSA for the in vitro and in vivo diagnosis and treatment of diseases [29]. In the present study, as illustrated in Figure 2, through using serum albumin as a delivery vector, the cell membrane-impermeable peptide AC3-I can be delivered into intracellular sites. Notably, cellular uptake significantly increased under hypoxia conditions compared to normoxia, gradually reaching saturation. This observation suggests that AC3-I@NPs have an enhanced permeability and retention (EPR) effect in ischemic tissue, which has also been reported in other studies [30]. While previous studies have explored diagnostic and therapeutic strategies for myocardial infarction using nanomaterials, our investigation marks the first examination of the membrane-impermeable peptide with HSA nanoparticles [5]. More importantly, we found that AC3-I@NPs exerted protective effects against cardiac injury in vitro and ex vivo without inducing cellular toxicity at the concentration we used, implying that AC3-I@NPs have great potential for use post cardiac infarction.

In addition, p-PLB, an endogenous substance, plays an important role in cardiomyocyte function [31]. Its phosphorylation contributes to the activation of CaMKII in cardiomyocytes. We demonstrated that treating cardiomyocytes with AC3-I@NPs inhibited CaMKII activation in both models of ischemic and electrical field pacing. Further investigation is warranted to elucidate the mechanism underlying the release of the AC3-I peptide from AC3-I@NPs, the behaviors underlying the peptide’s binding to CaMKII to inhibit CaMKII activation, and its distribution inside cardiomyocytes. More importantly, the protective effects of AC3-I@NPs against cardiac injury were confirmed in vitro and ex vivo without inducing cellular toxicity at the concentration we used.

The pH in the tissue environment is a critical factor influencing drug release behavior in nanoparticle medicine. While our study focused on cardiac samples, further research exploring the release of AC3-I at different pH levels is warranted, given the variations expected between ischemia–reperfusion and physiological conditions. Additionally, further investigation is necessary to validate our findings in animal studies and confirm the protective effects of AC3-I@NPs against cardiac injury in vivo, building upon the in vitro and ex vivo evidence presented here.

## 5. Conclusions

In summary, the current work reports the development of nanoparticles containing the cell membrane-impermeable CaMKII inhibitor AC3-I. The prepared nanoparticles exhibited favorable stability and physicochemical characteristics. Investigations into cellular uptake revealed a notable augmentation in the uptake of AC3-I@NPs by rat cardiac myocytes under hypoxic conditions compared to normoxic conditions. The therapeutic effects of nanoformulations on cellular injuries induced by hypoxia–reoxygenation in cardiomycytes and ischemia–reperfusion in isolated hearts were also investigated. AC3-I@NPs demonstrated significant protective effects against cardiac injury both in vitro and ex vivo while maintaining cellular integrity without inducing toxicity. This novel formulation has great potential for use in further clinical applications of the CaMKII inhibitor AC3-I in the treatment of ischemic heart diseases.

## Data Availability

Data are available from the corresponding author upon request.

## Abbreviations

ARCMs	Adult rat cardiomyocytes
CCM	Curcumin
CK	Creatine kinase
CaMKII	Ca^2+^/calmodulin-dependent protein kinase II
EFS	Electric field stimulation
H/R	Hypoxia–reoxygenation
HSA	Human serum albumin
I/R	Ischemia–reperfusion
LDH	Lactate dehydrogenase
MI	Myocardial ischemia
PBS	Phosphate-buffered saline
PLB	Phospholamban
ROS	Reactive oxygen species
RES	Reticular endothelial system
Tris	Tris(hydroxymethyl)aminomethane
